# 5 years of experience with a large-scale mentoring program for medical students

**DOI:** 10.3205/zma000947

**Published:** 2015-02-11

**Authors:** Severin Pinilla, Tanja Pander, Philip von der Borch, Martin R. Fischer, Konstantinos Dimitriadis

**Affiliations:** 1 Ludwig-Maximilians-University Munich, Department of Neurology, Munich, Germany; 2 Ludwig Maximilians-Universität Munich, Institut für Didaktik und Ausbildungsforschung in der Medizin, Munich, Germany; 3 Ludwig Maximilians-Universität Munich, Medizinische Klinik and Poliklinik IV, Munich, Germany

**Keywords:** Mentoring, undergraduate medical students, evaluation, online matching

## Abstract

In this paper we present our 5-year-experience with a large-scale mentoring program for undergraduate medical students at the Ludwig Maximilians-Universität Munich (LMU). We implemented a two-tiered program with a peer-mentoring concept for preclinical students and a 1:1-mentoring concept for clinical students aided by a fully automated online-based matching algorithm. Approximately 20-30% of each student cohort participates in our voluntary mentoring program. Defining ideal program evaluation strategies, recruiting mentors from beyond the academic environment and accounting for the mentoring network reality remain challenging. We conclude that a two-tiered program is well accepted by students and faculty. In addition the online-based matching seems to be effective for large-scale mentoring programs.

## Zusammenfassung

In diesem Artikel werden die Erfahrungen mit der Implementierung und Organisation eines Mentorenprogrammes für eine große Anzahl Medizinstudierender an der Ludwig-Maximilian-Universität München (LMU) der letzten fünf Jahre vorgestellt und diskutiert. Aufgrund einer Bedarfsanalyse unter Studierenden der medizinischen Fakultät der LMU implementierten wir ein zweigeteiltes Mentorenprogramm mit einem Peer-Mentoringkonzept für Studierende der Vorklinik und einem individuellen 1:1-Mentoringkonzept für Studierende der Klinik, welches durch einen online-basierten Matching-Algorithmus unterstützt wird. 20-30% aller Medizinstudierenden der LMU München nehmen freiwillig an unserem Mentorenprogramm teil.

Zu den speziellen Herausforderungen gehören unter anderem die Entwicklung eines longitudinalen Evaluationskonzeptes, die Gewinnung von Mentoren auch über Universitätskliniken hinaus, und die Abbildung eines Mentoren-Netzwerks im Rahmen eines formalen Mentorenprogrammes.

Ein zweigeteiltes Programm mit online-basiertem Matching scheint ein effektives Konzept für groß angelegte Mentorenprogramme zu sein und ist unter Medizinstudierenden und an der medizinischen Fakultät gut akzeptiert worden.

## Authorship

Pinilla Severin and Pander Tanja contribute equally to the paper.

## Introduction

### What we know

In the light of comprehensive competence catalogues, medical students face substantial challenges in finding their individual professional path. With a continuously high workload and multiple career opportunities, medical students have a marked demand for additional support [1]. Mentoring as a powerful educational tool [4] seems to be effective for key issues in medical students’ professional development and support [3]. The World Federation for Medical Education names structured and formal mentoring programs as a standard of basic medical education [http://www.wfme.org/standards/bme citited 05.02.14]. Although academic mentoring is acclaimed and perceived as a successful support tool and career catalyst, there is a lack of formal mentoring programs in many countries [6]. In Germany there are several mentoring programs for medical students but the overall availability of mentoring is still poor [11]. Longitudinal data on the impact of formal mentoring programs is still lacking [13].

In this report, we present experience with the last five years of our large-scale mentoring program for medical students at LMU Munich. We provide an overview of the initial implementation plan and the current state of affairs together with selected program outputs. Finally we discuss challenges and potential implications for practice.

## Project discription

### What we did 

The vision of our mentoring program was and is to provide every student of the medical faculty at LMU Munich (n>4000) with the opportunity to find a suitable support for their individual needs, for example by choosing a mentor or participating in relevant events. Intended short-term outputs included students’ positive perception of faculty-based support and the chance to realize the students’ individual ideas of professional self-development. We also aimed at an extended and improved inner-faculty networking structure and learning culture as long-term outcomes.

The mission of our mentoring program consisted of three main aspects:

1. To strengthen the horizontal (within one semester) and vertical network (above all hierarchical and educational levels) of the faculty and thus provide an efficient support structure.
2. To empower medical students and strengthen students’ participation in faculty life for example by organizing and participating at events, which support the global and professional development of a modern physician. The desired competencies of a modern physician include the roles as a health advocate, a collaborator, a manager, a communicator, a scholar and a professional [5].
3. To foster the scholarly approach to mentoring activities by a broad research activity and regularly evaluation of all events and the mentoring activities.

The medical curriculum at LMU Munich consists of two pre-clinical and four clinical years. Initially we conducted a needs analysis among medical students in all semesters (n=578, response rate=14.1%). We identified an overall demand for support through the faculty, with an emphasis on personal and professional development as well as career planning [15]. We consequently launched a two-tiered mentoring program (MeCuM-Mentor) consisting of a 1:1-mentoring program for clinical students and a peer-mentoring program with a focus on preclinical students. The students’ and mentors’ participation at the program is voluntary.

In the 1:1-mentoring program all students in the clinical years are given the opportunity to match with an individual mentor. The mentors are voluntarily participating physicians with different backgrounds and from different disciplines and specialties. 

In our program, students can either

1. freely choose their mentors among faculty members they come in contact with and register this relationship on our website or
2. choose from a preselected group of online-matched mentors with similar personal (e.g. gender, age, sport and cultural preferences) and professional interests (e.g. research activities, specialties, field, USMLE) [15].

The majority of mentoring-relationships in our program are formed through the online matching option (88.2%), where students and physicians complete online profiles consisting of 13 items with regards to their professional background and career interests as well as to their work-life priorities. Responses are given using a 6-level Likert scale and a free text section. A computer-based matching score using a weighted correlation algorithm presents students with the 10 mentors who are most likely to suit their profile [14]. 

Since this year we implemented two additional ways of matching a mentor. Students have the opportunity to search freely all profiles of registered mentors at our website by using individual search terms or have the opportunity to get a personal counsel from our team.

After successful matching the mentee and the mentor meet personally, become acquainted and agree on personal goals and meet as needed. One of the most discussed topics between mentees and mentors is career planning and mentees mostly perceive their mentors as a counselor. If either the mentee or the mentor chooses to end the relationship, the student can re-match at any time [3], [15].

The peer-mentoring program was initially designed primarily for pre-clinical students but students from clinical semesters regularly participated in most peer-mentoring events as well. The driving momentum of the peer-mentoring program originates from motivated students from all semesters, so called junior mentors. The individual junior mentors are selected through a democratic selection process. 

After election, junior mentors are invited to a two-day introductory seminar with workshops on mentoring, leadership and team building to prepare for their role as multiplicators of the mentoring idea. Junior mentors are elected on a yearly basis and represent contact persons and mentors for their fellow students. In addition junior mentors organized a range of mentoring events.

All data used in our results is generated from our website or from individual evaluation of events.

## Results

### What we do

Through the continuous political support of the deans of the medical school, MeCuM-Mentor was successfully integrated in the medical faculty as a formal project of the Institute for Medical Education at the LMU Munich. Currently the coordinating team consists of two full-time employees, one with a pedagogical background and another with a medical background. Additionally, four to six student assistants are involved in organizing and evaluating the peer-mentoring events throughout the academic year. The program is predominantly funded through tuition fees and through event-specific sponsors.

The central information source for all stakeholders is a webpage (http://www.mecum-mentor.de) which consists of both publicly accessible resources as well as password secured internal sites to administer mentor and mentee profiles and mentoring relationships. In 2013 the webpage was visited roughly 14,000 times.

We also use social media (primarily Facebook) as well as newsletters from the student government body and self-designed posters to announce any upcoming events or updates that are relevant in the context of mentoring and networking.

In order to recruit new mentors, we introduce the program every October in all clinical departments associated with our medical school. Furthermore we organize a mentoring event (MentoringFest) once a year, which includes an introductory lecture to mentoring, a mentoring speed dating market as well as mentoring round-tables in order to facilitate participation in the mentoring program as well as meeting potential mentors from all hierarchical levels in person. 

**Figure 1:**
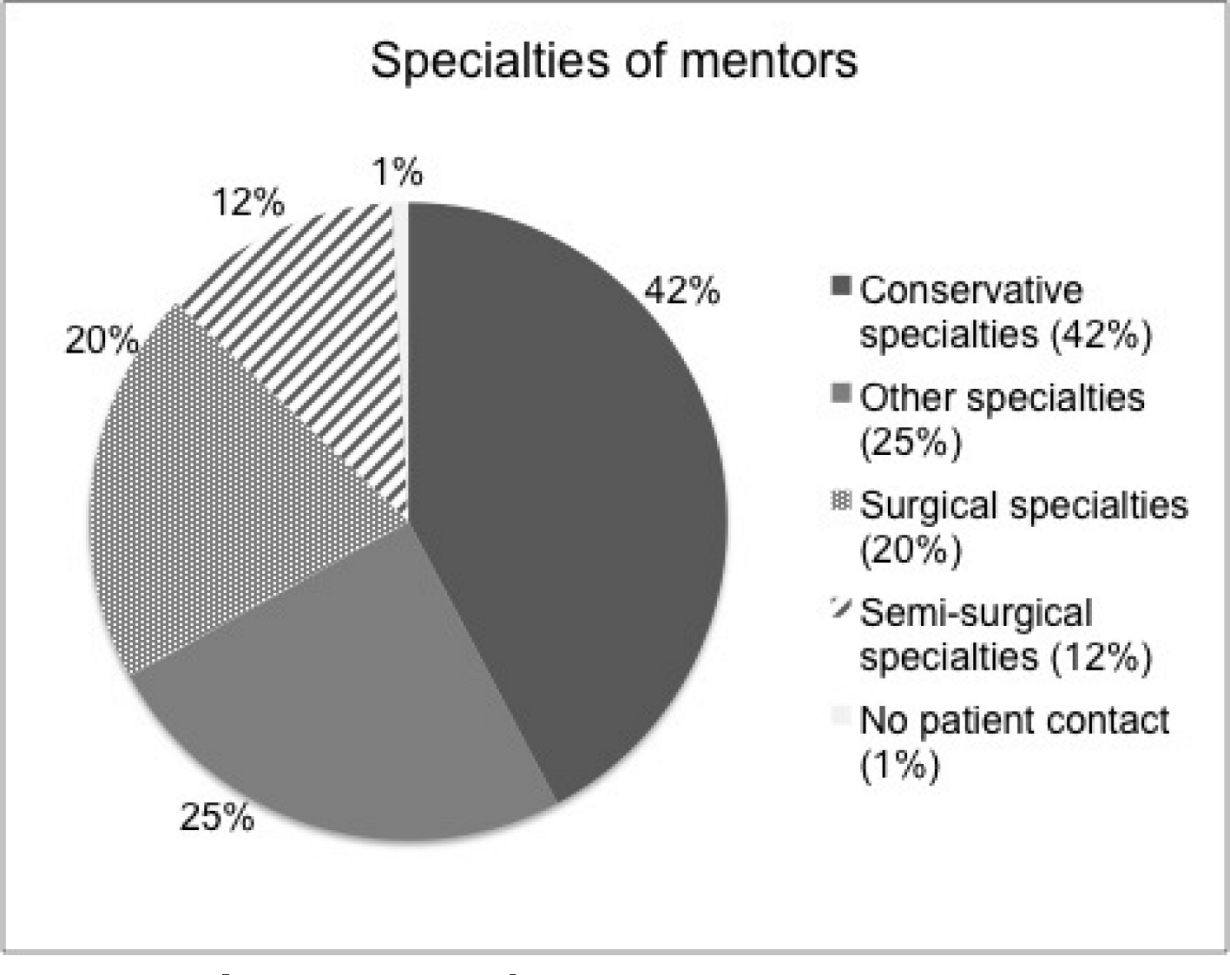
Specialties of mentors participating in the 1:1-mentoring program for clinical students. ‘Other specialties’ include anesthesiology, radiology and pathology. ‘No patient contact’ refers to medical doctors working in the industry.

Approximately 399 mentors are currently active in our program. 187 of them have still available places for mentees. On average one mentor has a formal mentoring-relationship with one to three mentees. 128 mentors are female and 271 mentors are male and they cover the full range of medical specialties (see Figure 1 (Fig. 1)). The majority of our mentors work within conservative medical specialties (42%). 32% are working in surgical or semi-surgical specialties. The remaining mentors are working in public health, medical education, industry or medical specialties with rare or no patient interaction. Mentees mostly perceived their mentors as counselors, providers of ideas, and role models [3]. As a preparation for their mission as a mentor, mentors can find supporting material on the webpage of the mentoring program. Also the mentoring office gives individual support for mentors, who have questions or concerns.

Approximately there are 3403 students registered at our website. 1440 of them are students of the clinical years of the medical curriculum Munich and are allowed taking part in the 1:1-mentoring. 842 out of this cohort have a mentor. 60% are female, which represents the students’ gender distribution at the medical faculty. They have an average age of 23.56±3.58 years and complete in average the 10.45±4.46 semester. The main topics, which are discussed in these 1:1-mentoring relationships, are the mentee’s personal goals, carrier planning and experiences abroad [3].

Mentees can find all information about the mentors and the vision and mission of the mentoring program at our website. Also our team answers all open questions and gives all the information a mentee needs by email, telephone or face-to-face.

**Figure 2:**
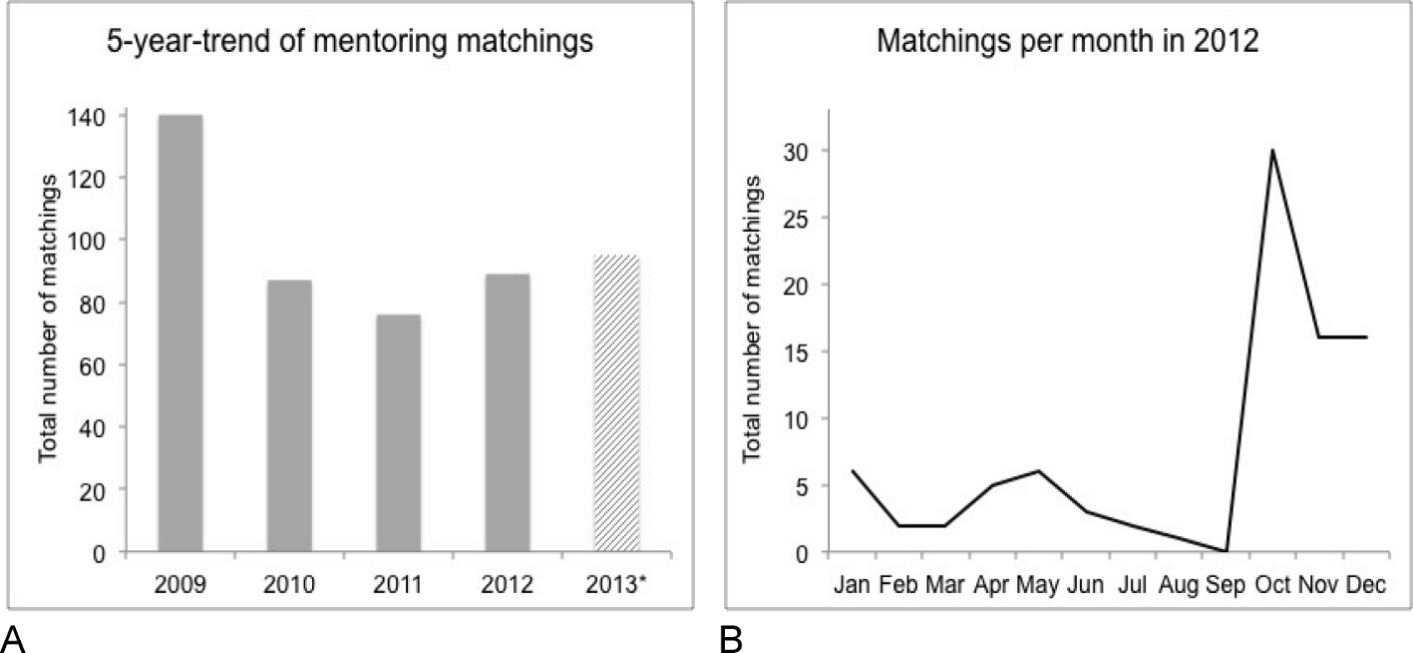
Total numbers of mentoring matchings over five years (Figure 2A) and per month in 2012 (Figure 2B). Matching is defined as successfully connecting a mentor with his or her mentee through an online matching algorithm.

The frequency and distribution of matchings over time are extracted from our mentoring database. Figure 2 shows the five-year trend of online-matched mentoring relationships (see Figure 2 (Fig. 2), Part A) and the matching trend within the academic year (see Figure 2 (Fig. 2), Part B). In 2008 (not shown in Figure 2 (Fig. 2)) as well as in 2009 the matching numbers were much higher because the program was newly introduced and students from all clinical semesters started matching at the same time. Based on these numbers we estimate that 20% to 30% of all students from each new student cohort entering the clinical phase (n≈400) participate in our voluntary mentoring program. The matching peaks in October and May are both consistent from 2009 until 2012 and reflect the beginning of the semesters. Students who proceed without any interruptions from high school to graduation, usually are part of the October cohort and thus might be more motivated in terms of career planning.

The junior mentor cohort currently consists of 38 students at different stages of their medical studies, including preclinical and clinical semesters. They were selected based on a written application with a short letter of motivation where they outline their plans to contribute to the peer-mentoring program during one academic year. General peer-mentoring events (including a student research conference ‘DoktaMed’ and a career-counselling discussion round ‘FacharztDuell’) together with more specific activities like an introductory clinical skills training (‘Fit for Famulatur’) or information sessions for high-stakes exams (‘Physikumsvorbereitung’) and orientations for the clinical years (‘Klinikeinführung’) are part of a diverse peer-mentoring project portfolio (see Table 1 (Tab. 1)). 

Every mentoring event organized by MeCuM-Mentor or by junior mentors is evaluated individually with a paper-based questionnaire. Most recent initiated activities start reaching beyond the medical school and include high-school students (‘Aufklärung gegen Tabak’). Some of those interventions have been evaluated more in-depth and have been published as reports or posters [7], [10], [16].

## Discussion

### What are the challenges?

Considering that mentoring is a complex construct and involves a myriad of sociocultural, professional and psychological elements, it is difficult to define a single-best outcome measure that would allow deciding whether a mentoring program is successful or not [12]. A particularly interesting and at the same time difficult to investigate component of mentoring is the effect of a mentoring relationship on the long-term professional development. There is a need for multi-centred prospective longitudinal studies that ideally enrol a whole generation of medical students following them through their career paths across different phases of undergraduate and graduate medical education, examining the influence of formal and informal mentoring on various outcome measures (for example: success, satisfaction, rectilinearity of the career path). Concerning the mentoring program at our faculty we currently cannot conclude what kind of impact it has from a long-term perspective. 

Another fundamental aspect refers to the characteristics of students who participate in a voluntary mentoring program. Based on our data, better performing students seem to be more likely to participate in a mentoring program [3]. Despite our efforts to address all students, we are still searching for adequate strategies to better include all clinical students in the 1:1-mentoring program or to specifically address diversity aspects and low-performing students as implemented in other programs [9].

Furthermore mentors who are active in our program are mostly working in an academic environment. In order to provide expertise and mentorship over the full range of professional development opportunities, additional recruiting strategies in order to reach practitioners and non-clinical mentors are needed. Additionally it is not clear yet, what the ideal way to select each junior mentor cohort would be. We initially tried a democratic election, however switched to a written application due to low election participation among undergraduate students and a relatively high workload of organizing student elections.

The focus of our mentoring program is on horizontal and vertical connection, sharing of experiences, building networks and facilitation of resource access. Other important aspects of mentoring, which draw on psychological counselling aspects or remediation techniques, might be underrepresented in our program since participating mentors do not have to fulfil specific criteria or go through additional training.

Some researchers have moved from conceptualizing mentoring as a dyadic relationship between two persons to a network of mentors, which might be closer to the reality of mentoring [2], [8]. However we find it challenging to translate this concept into a formalized mentoring program and to reflect the informal mentoring relationships, which constantly are being formed through research internships, clinical electives or accidental acquaintances. It might also not be necessary if the point of a mentoring program is to provide medical students with a first entry point to actively engage with and work on their career and personal goals.

## Conclusions

### What are the implications for practice? 

#### Individual level

The voluntary participation of mentors and junior mentors is an essential part of our program. We are convinced that intrinsic motivation is condition as well as foundation of a programs’ success. However, formal recognition of mentoring activities might additionally increase participation rates and foster a mentoring culture within an institution or faculty.

Mentoring programs do not function without continuous personal input. Despite a well-established online platform we regularly visit morning case discussions, lunch conferences and students’ lectures to inform faculty members about the benefits of participating in the program and being part of the mentoring culture at our faculty. We also established an open-door-policy in our office to provide all stakeholders of the mentoring program with a physical reference and support point.

From the regularly organized peer-mentoring events with continuously high attendance rates we conclude that our junior mentor concept is successful in terms of leveraging the multiplicator potential of motivated students. Positive overall evaluation results support this observation.

##### Program level

The two-tiered mentoring concept with an online matching algorithm seems to be a suitable solution for large-scale mentoring programs. It allows mentoring for high numbers of students by simultaneously ensuring the quality of individual support [3]. We are convinced that investing personal and financial resources in a content-management system is worthwhile, since it harbors a lot of the mentoring program mechanics, which includes registration, matching, internal and external communication. If we only had an informative website and did all the rest manually, the time and effort would dramatically increase. Still the amount of time and effort that needs to be spent on keeping a webpage up-to-date should not be underestimated.

At the moment there seems to be no consensus on what kind of matching process would be ideal for a mentoring program in medical school. From our experience an online matching program seems to be comparable to sympathy-based accidental and informal mentoring relationships. We assume that through our extended matching algorithm there is a potentially higher satisfaction rate as compared to random matching of mentors and mentees, although we have no data at this point to satisfactorily test this hypothesis. We suggest that future studies use controlled trials to compare measurable outcomes, such as mentoring satisfaction, frequency of interaction between mentor and mentee and duration of mentoring relationship in order to provide better recommendations for how the matching process should be designed.

##### Faculty level

Depending on the financing and hierarchical structure of a mentoring program it is important to effectively collaborate with different departments and student association bodies in order to have the necessary political support from a long-term perspective. Official support from decision-makers and stakeholders is also crucial in this context. We found the support from well-regarded active and retired professors and deans as catalyzing for launching the mentoring program as well as for initiating new mentoring activities.

Finally, an effective and efficient mentoring program can also function as a catalyst for medical education innovations. The aggregated expertise and experience in organizing events from small informal gatherings to communication and clinical skills courses as well as student research conferences can serve as a continuous source to foster educational innovation and scholarship.

#### What is the conclusion? 

The five-year experience with our mentoring program shows that a large-scale mentoring program could be successfully integrated in the medical faculty of the LMU Munich. A two-tiered mentoring concept, with individual mentors for clinical semesters and junior mentors, works successfully for faculties with high numbers of students. Intrinsic motivation of the mentors and formal supportive structures are crucial. The presented voluntary mentoring concept is being used by roughly every fourth student of our faculty and should be transferable to other faculties and disciplines. 

Longitudinal and multi-centered research projects are needed to better estimate the effects of mentoring relationships on individual career paths and career satisfaction as well as on personal and professional network development and work-life-balance. Innovations in communication technology and use of social media might also change the scope of mentoring activities and provide additional opportunities to effectively mentor medical students.

## Acknowledgement

We would like to thank Sylvère Störmann and Stefan Galster for their outstanding support with programing the web-based components of MeCuM-Mentor.

## Competing interests

The authors declare that they have no competing interests.

## Figures and Tables

**Table 1:**
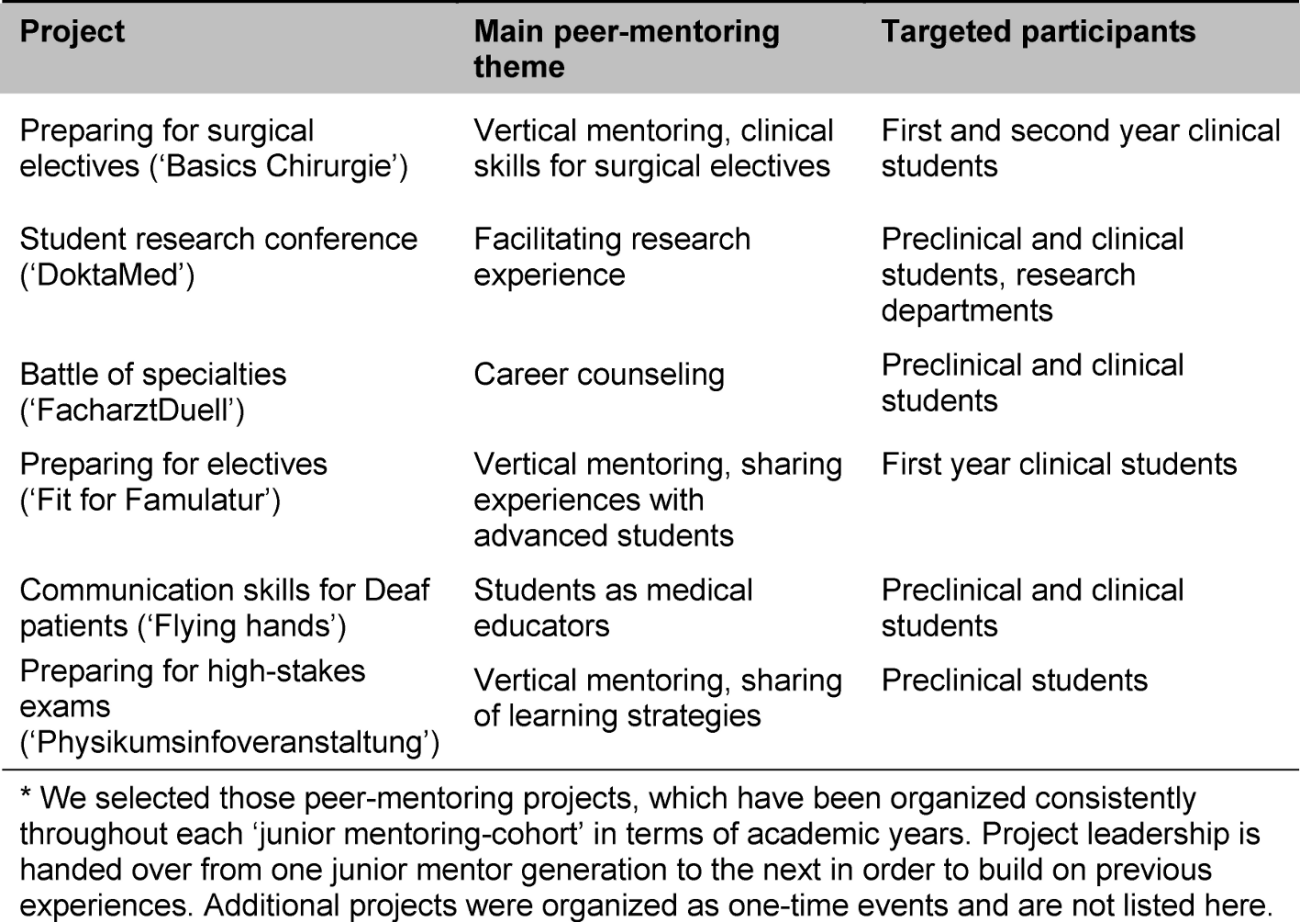
Peer-mentoring project portfolio*
